# Refractory hypoxemia caused by hepatopulmonary syndrome: a case report

**DOI:** 10.1186/1752-1947-8-418

**Published:** 2014-12-10

**Authors:** Morgen L Govindan, Kevin W Kuo, Maryam Ghadimi Mahani, Thomas P Shanley

**Affiliations:** 17 Department of Pediatrics and Communicable Diseases, C.S. Mott Children’s Hospital, University of Michigan, 1500 East Medical Center Drive, Ann Arbor, MI USA; 18 Department of Radiology, C.S. Mott Children’s Hospital, University of Michigan, 1500 East Medical Center Drive, Ann Arbor, MI USA

**Keywords:** Contrast echocardiography, Hepatopulmonary syndrome, Pediatric, Refractory hypoxemia

## Abstract

**Introduction:**

Hepatopulmonary syndrome is a clinical syndrome that can affect patients of all ages with liver disease and is more common in children with biliary atresia. Contrast echocardiography is the test of choice to diagnose the presence of intrapulmonary vascular dilatation. The established treatment for hepatopulmonary syndrome is liver transplantation.

**Case presentation:**

We present the case of an 8-month-old Caucasian baby boy with a history of biliary atresia, polysplenia, and interrupted inferior vena cava who presented with hypoxemia and cyanosis that progressed rapidly. A chest computed tomography angiogram revealed significant dilatation of the pulmonary vasculature, prompting further evaluation and diagnosis of hepatopulmonary syndrome with contrast echocardiography. He was maintained on a milrinone infusion while awaiting liver transplantation. His hypoxemia improved slowly following liver transplantation, requiring tracheostomy and prolonged ventilator dependence.

**Conclusions:**

Hepatopulmonary syndrome should be included in the differential for progressive hypoxemia in children with liver disease, particularly those with biliary atresia. Imaging with chest computed tomography angiogram and contrast echocardiography should be considered in cases of unexplained refractory hypoxemia.

## Introduction

Hepatopulmonary syndrome (HPS) is a rare condition in children, although it is more common in children with a history of biliary atresia [1, 2]. HPS is identified by the presence of liver disease and intrapulmonary vascular dilatation (IPVD) resulting in impaired oxygenation. Clinical manifestations of HPS are related to both liver and pulmonary disease and include spider nevi, clubbing, dyspnea, and cyanosis [3].

Currently, the only proven treatment for HPS is orthotopic liver transplantation (OLT), and HPS is considered a relative indication for transplantation [4]. In fact, because HPS may present in the absence of synthetic liver dysfunction, it may be the basis for requesting a petition of exception from the United Network for Organ Sharing (UNOS). Here, we describe a case of a baby with heterotaxy syndrome and biliary atresia who presented with progressive hypoxemia. The diagnosis of HPS was suggested based on findings noted on a chest computed tomography (CT) angiogram. The baby was maintained on a therapeutic regimen including a milrinone infusion until OLT could be performed, after which his hypoxemia gradually resolved. To the best of our knowledge, the use of milrinone in a pediatric patient with HPS has not been previously described. The diagnosis, management, and outcomes associated with HPS are reviewed.

## Case presentation

An 8-month-old Caucasian baby boy with heterotaxy syndrome was admitted to our general pediatric ward after presenting to a scheduled clinic visit with fever, hypoxemia and cyanosis. His history was significant for dextrocardia, polysplenia, interrupted inferior vena cava, biliary atresia status post-Kasai procedure, and malrotation status post-Ladd’s procedure. He was febrile on presentation, and was started on oxygen via nasal cannula for oxyhemoglobin saturations as low as 70%. His chest radiograph on admission showed a patchy right lower lobe opacity, and he was started on antibiotics out of concern for pneumonia. He was subsequently transferred to our pediatric intensive care unit (PICU) for refractory hypoxemia requiring high flow oxygen therapy.

Throughout his PICU admission, he continued to require high flow oxygen up to 9L per minute and fraction of inspired oxygen (FiO_2_) of 1 to maintain oxyhemoglobin saturation greater than 75%, with persistent desaturations while upset. Early in his admission, he developed increased work of breathing requiring positive pressure ventilation with bilevel positive airway pressure. A chest radiograph revealed pulmonary edema, and brain natriuretic peptide (BNP) at that time was elevated at 1130pg/mL. His pulmonary edema improved with diuresis, resulting in de-escalation of therapy with return to standard nasal cannula. He required blood pressure support with a dopamine infusion during aggressive diuresis, which was subsequently weaned off without complication. Multiple echocardiograms revealed no clear cardiac etiology of his hypoxemia.

**Figure 1:**
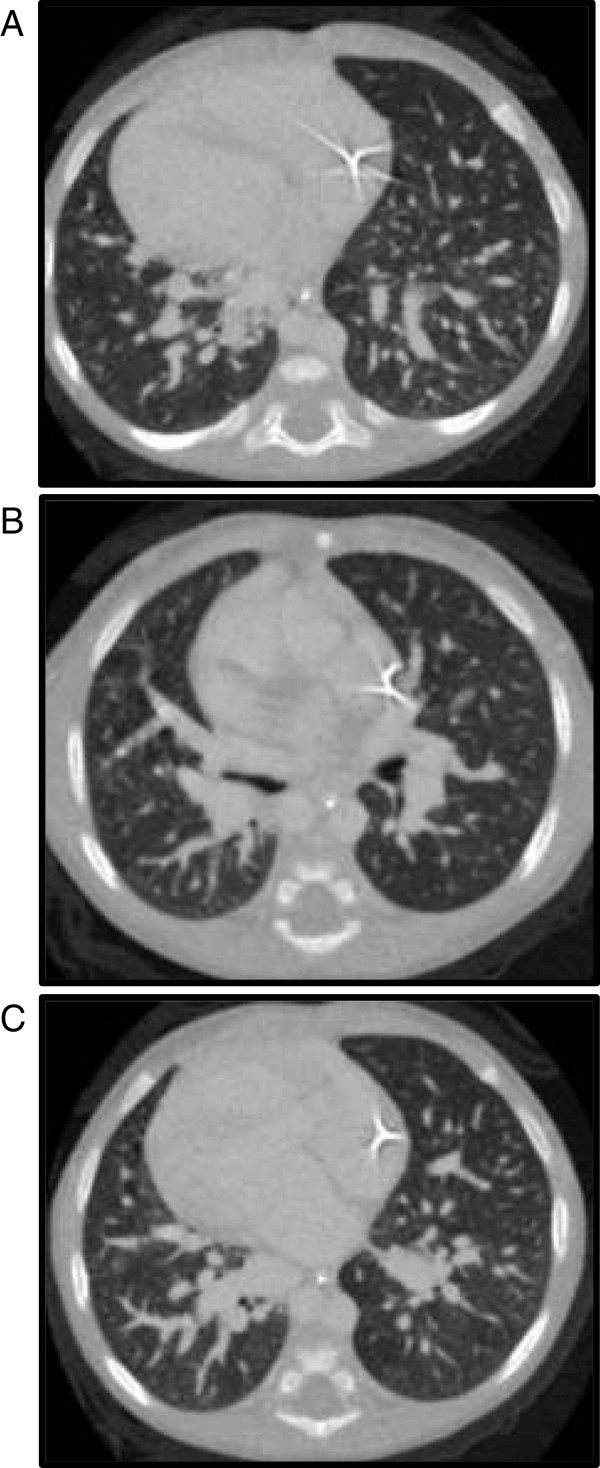
**Axial computed tomography angiography images using intravenous contrast material in an 8-month-old baby boy below the level of carina (A) towards the base (B, C).** Diffuse dilatation of the pulmonary vasculature is seen to be more prominent in caudal sections **(B, C)**. Lung parenchyma is normal and dextrocardia is seen.

**Figure 2:**
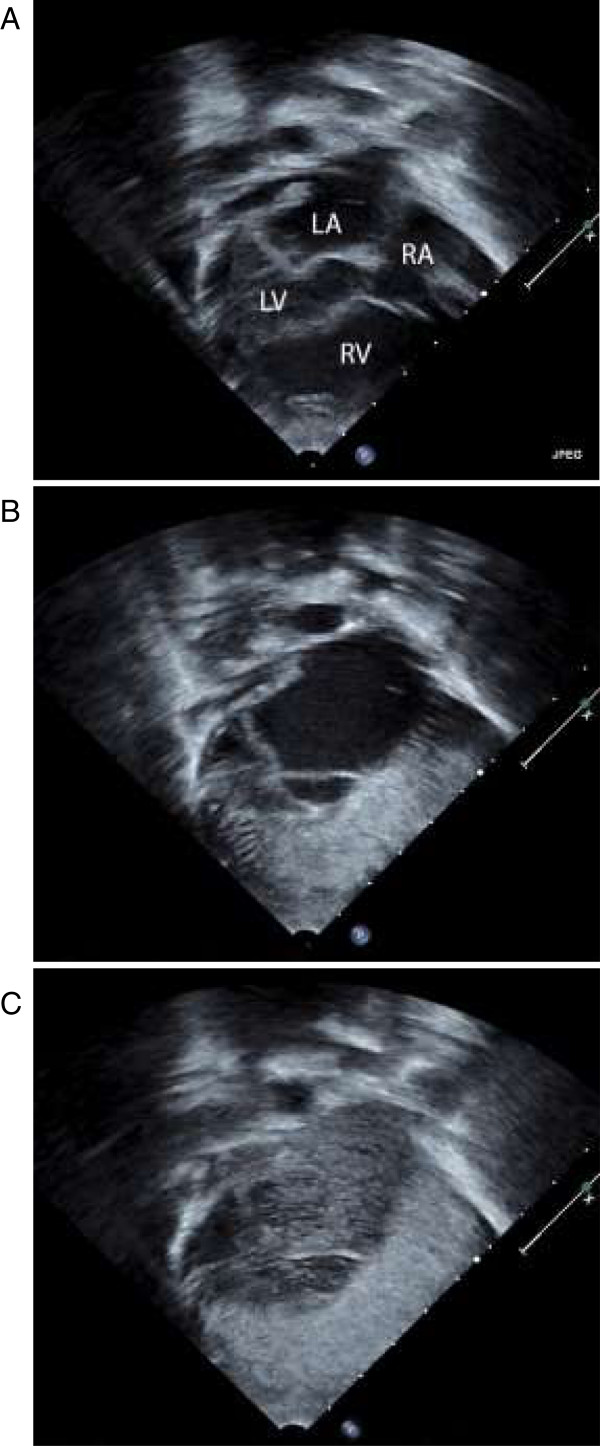
**Off-axis apical view with contrast injection (agitated saline).** Patient has dextrocardia **(A)**. Note good opacification of the right atrium and right ventricle in **(B)** with contrast. No contrast is seen initially in the left atrium, suggesting no atrial level shunt. Bubbles were seen in the left atrium after several beats **(C)** suggesting intrapulmonary shunts or arteriovenous malformations rather than intracardiac shunts (images courtesy of Jimmy C Lu MD). Abbreviations: LA, left atrium; LV, left ventricle; RA, right atrium; RV, right ventricle.

A chest CT angiogram with high resolution reconstruction of the lung parenchyma was performed on the recommendation of our Radiology department to further evaluate the pulmonary vasculature and lung parenchyma. CT imaging showed dilatation of the pulmonary vasculature bilaterally without significant abnormalities of the lung parenchyma (Figure 1). Pulmonary arterial hypertension was felt to be an unlikely cause of his dilated pulmonary vasculature as right ventricular pressures were measured as less than half of systemic pressures on echocardiogram. Given his history of biliary atresia and constellation of symptoms, HPS was included on the differential diagnosis. He underwent agitated saline contrast study, which showed bubbles entering the right-sided atrium (systemic atrium in the setting of dextrocardia) through a right pulmonary vein approximately 3 cardiac beats after opacification of the left-sided chambers (Figure 2), consistent with clinically significant intrapulmonary shunting.

Abdominal magnetic resonance imaging (MRI) was performed to evaluate for the presence of a congenital portosystemic shunt given the strong association of Abernethy malformation and heterotaxy syndrome and the possibility of reversal of HPS with shunt occlusion. MRI did not reveal portosystemic shunting. The patient subsequently underwent evaluation for liver transplantation. His liver enzymes and coagulation studies were normal. His bilirubin levels were also normal and given normal bile ducts on abdominal ultrasound, a hepatobiliary iminodiacetic acid scan was not performed. Abdominal ultrasound also demonstrated diffuse liver disease, patent portal vein with hepatopetal flow, and no ascites. He did have polysplenia with splenomegaly. He had no signs of collateral circulation on examination. No abnormalities were noted in his stools. His partial pressure of oxygen in arterial blood (PaO_2_) was measured at 29mmHg while on FiO_2_ of 0.5. He was listed for transplant with a pediatric end-stage liver disease score of 28, which was further increased to 35 following petition to UNOS for worsening clinical status.

Prior to diagnosis of HPS, the patient was initiated on a milrinone infusion for suspicion of diastolic heart failure. Following the diagnosis, he was trialed off milrinone with the rationale that further pulmonary vasodilation actually may be deleterious given the diagnosis of HPS. However, he responded poorly with subsequent oxyhemoglobin desaturations requiring reinitiation of the infusion. His oxygenation stabilized on a milrinone infusion of 0.5μg/kg/minute. Inhaled nitric oxide was also trialed without significant improvement in oxygen requirement and was subsequently weaned off. Oxygen requirements ultimately stabilized on a diuretic regimen including spironolactone and furosemide.

**Figure 3:**
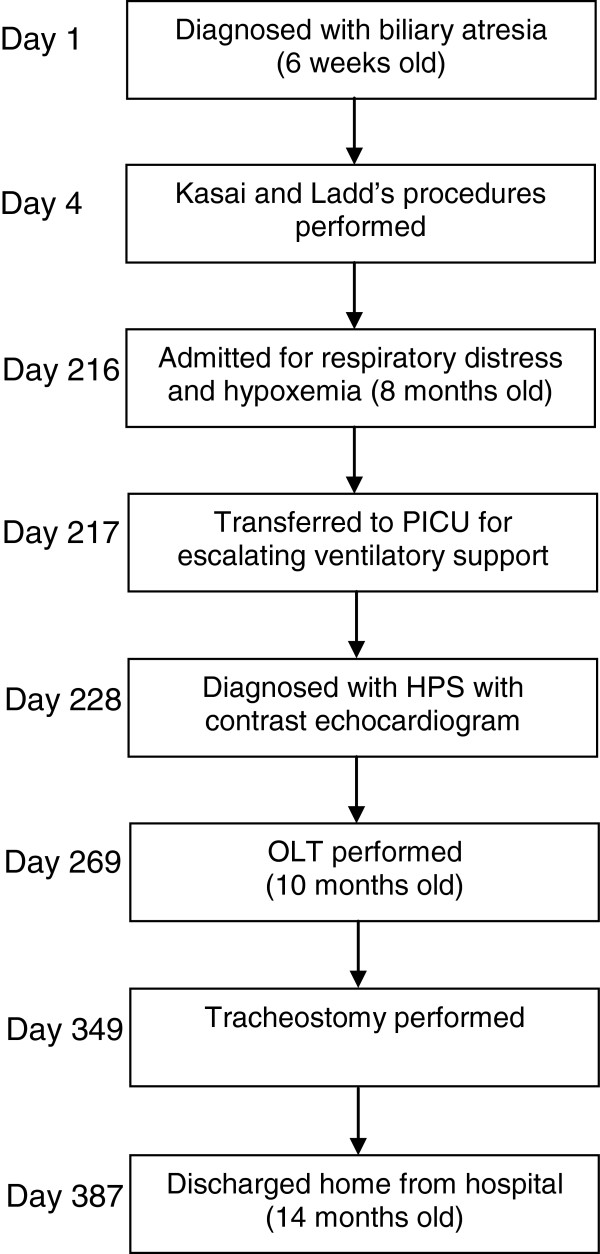
**Timeline of the patient’s clinical course.** Abbreviations: HPS, hepatopulmonary syndrome; OLT, orthotopic liver transplantation; PICU, pediatric intensive care unit.

He underwent OLT 2 months after initial presentation. His postoperative period was complicated by hypercarbic and hypoxemic respiratory failure requiring mechanical ventilation. He underwent tracheostomy for chronic ventilator-dependent respiratory failure. He was transferred out of the PICU 2 months after OLT and subsequently weaned off mechanical ventilation during the daytime. He was discharged home 4 months following OLT on FiO_2_ of 0.21 through a tracheostomy mask with nighttime ventilator support. He continues to do well. Please see Figure 3 for a timeline of the patient’s clinical course.

## Discussion

This case report of HPS highlights the importance of considering HPS in patients with liver disease and otherwise unexplained hypoxemia, even without significant liver dysfunction. It also highlights the diagnostic modalities used to diagnose HPS in pediatric patients including CT angiogram and contrast echocardiography. Given the pathophysiology of HPS involving pulmonary vascular dilatation, therapies such as milrinone and nitric oxide may appear counterintuitive. This is a limitation of our approach as it is unclear why our patient had such a significant response to milrinone, although we speculate there may have been a component of diastolic heart failure given the elevated BNP level.

The clinical diagnosis of HPS is based upon the triad of IPVD, liver disease, and impaired oxygenation, defined as partial pressure of oxygen <80mmHg or an alveolar-arterial oxygen gradient greater than 15mmHg when breathing room air [5]. HPS is a rare condition that can affect patients of all ages. The patient in this case is notable for his young age; however, HPS has been diagnosed in children as young as 6 months [1]. It is associated with biliary atresia and polysplenia, and the reported prevalence of HPS in children with liver disease has ranged from 0.5% in patients with portal vein thrombosis to 9% to 20% in those with biliary atresia [1, 2].

Common presenting signs and symptoms of HPS include dyspnea, cyanosis and hypoxemia, which may worsen with crying and exertion. Platypnea (dyspnea that worsens in the upright position compared to supine) and orthodeoxia (decrease in PaO_2_ >5% or 4mmHg in the upright position) are characteristic findings in HPS, and may be related to increased pulmonary shunting in the context of altered pulmonary vascular tone [6]. Of importance, HPS may be the cause of otherwise unexplained hypoxemia in a patient with acute or chronic liver disease, as was seen in this case. Other signs and symptoms of HPS are related to liver disease, such as spider nevi, palmar erythema, and thenar atrophy [3]. Spider nevi are cutaneous vascular lesions that have been associated with more severe hypoxemia and pulmonary dilatation and have thus been proposed as a cutaneous marker for IPVD [7].

With regard to abnormalities in the results of laboratory tests, elevated BNP has been found in children with intrapulmonary vascular shunting, which is hypothesized to reflect the increased left atrial volume in patients with IPVD [8]. Of interest, the presence of intrapulmonary shunting is not associated with the severity of liver disease, as was demonstrated in this patient who had HPS in the absence of synthetic liver dysfunction [9].

From a radiological standpoint, HPS is characterized by diffuse dilatation of the peripheral pulmonary vasculature. In this case, the diagnosis of HPS was first discussed following the findings of dilated pulmonary vasculature on CT angiogram. Thus, CT angiogram can be helpful in a patient with liver disease and unexplained hypoxemia. Subsequent testing via contrast-enhanced transthoracic echocardiography with agitated saline is useful in detecting the presence of IPVD. During this procedure, saline is shaken to produce microbubbles greater than 10μm in diameter and administered through a peripheral vein in the arm. The presence of microbubble opacification in the left atrium within 3 to 6 cardiac cycles following opacification of the right atrium is considered a positive test for the presence of intrapulmonary vascular shunting [5]. In consequence, contrast echocardiography is the recommended study for the evaluation of IPVD in diagnosing HPS, as it is more sensitive than the lung perfusion scan with technetium and has no radiation exposure.

Of note, it is important to exclude a congenital extrahepatic portosystemic shunt (Abernethy malformation) in children with HPS, especially in patients with heterotaxy syndrome, as this will impact the management of the patient. In the presence of a portosystemic shunt, HPS may reverse with shunt occlusion if the portal vein is patent. If an extrahepatic portosystemic shunt has been missed, hypoxemia may return after the initial response to liver transplantation [10].

At this time, there are no proven pharmacological treatments for HPS, although there are case reports of improvement with therapies aimed at inhibiting nitric oxide [11]. Pentoxifylline (PTX), a phosphodiesterase inhibitor, is one such therapy that has been used in studies for the treatment of HPS. The potential of PTX in HPS has been attributed in part to its inhibitory effect on nitric oxide synthase resulting in decreased production of nitric oxide. PTX has also been found to down regulate angiogenesis [11]. In a study performed by Kianifar *et al.*, pediatric patients exhibited significant increases in arterial oxygen pressure, oxyhemoglobin saturation, and decreased alveolar-arterial oxygen gradient following 3 months of treatment with PTX [12]. Milrinone is a selective phosphodiesterase 3 inhibitor with positive inotropic and vasodilatory effects. Although implemented as a bridge in our patient with some success, the use of milrinone has not been well studied in HPS. Nonetheless, milrinone has been used as rescue therapy for portopulmonary hypertension at the time of OLT [13].

Liver transplantation remains the only established therapy for HPS. Several studies have shown the reversibility of HPS in the majority of pediatric patients following OLT [2]. Post-transplantation survival has been found to be lower in patients with HPS compared to overall post-OLT survival, with 1-year post-transplantation survival ranging from 62% to 84% in patients with HPS compared to 90% to 92% in patients without HPS [9, 14]. Specifically in pediatric patients with HPS, the 1-year survival following OLT ranges from 62% to 100% [2, 15]. Gupta *et al.* found no significant difference in post-transplantation survival between pediatric patients with and without HPS; however, all patients with HPS in the study had PaO_2_ >50mmHg [2]. It should be noted that preoperative hypoxemia is an important prognosticator for both postoperative mortality and time to resolution of hypoxemia following transplant in HPS [14, 15]. Hypoxemia may transiently worsen postoperatively, requiring prolonged support with mechanical ventilation for up to several months following transplant. The patient in this case was severely hypoxemic preoperatively and exhibited prolonged ventilator dependence after OLT.

## Conclusions

In summary, HPS is characterized by progressive hypoxemia caused by intrapulmonary shunting in patients with liver disease. While it is a rare condition in the general population, it is not uncommon in patients with biliary atresia, and should thus be included in the differential diagnosis of hypoxemia in children with biliary atresia, even without evidence of synthetic liver dysfunction. Saline contrast echocardiogram and CT angiogram may be useful studies in such patients and typically reveal extracardiac shunt and pulmonary vascular dilatation in those with HPS. Full resolution of HPS is possible with liver transplantation, and the severity of hypoxemia is associated with higher postoperative mortality. Therefore, the diagnosis of HPS should ideally be made as early as possible.

## Consent

Written informed consent was obtained from the patient’s mother for publication of this case report and any accompanying images. A copy of the written consent is available for review by the Editor-in-Chief of this journal.

## Abbreviations

BNP: Brain natriuretic peptide
CT: Computed tomography
FiO_2_: Fraction of inspired oxygen
HPS: Hepatopulmonary syndrome
IPVD: Intrapulmonary vascular dilatation
MRI: Magnetic resonance imaging
OLT: Orthotopic liver transplantation
PaO_2_: Partial pressure of oxygen in arterial blood
PICU: Pediatric intensive care unit
PTX: Pentoxifylline
UNOS: United Network for Organ Sharing.
